# Effects of exposure to elevated temperature and different food levels on the escape response and metabolism of early life stages of white seabream, *Diplodus sargus*

**DOI:** 10.1093/conphys/coac023

**Published:** 2022-05-06

**Authors:** João Almeida, Ana Rita Lopes, Laura Ribeiro, Sara Castanho, Ana Candeias-Mendes, Pedro Pousão-Ferreira, Ana M Faria

**Affiliations:** MARE - Marine and Environmental Sciences Centre, ISPA, Instituto Universitário, 1149-041, Lisbon, Portugal; MARE - Marine and Environmental Sciences Centre, ISPA, Instituto Universitário, 1149-041, Lisbon, Portugal; MARE - Marine and Environmental Sciences Centre, Faculdade de Ciências da Universidade de Lisboa, 8700-194, Lisbon, Portugal; Portuguese Institute for the Ocean and Atmosphere - IPMA, Aquaculture Research Station, 1749-016, Olhão, Portugal; Portuguese Institute for the Ocean and Atmosphere - IPMA, Aquaculture Research Station, 1749-016, Olhão, Portugal; Portuguese Institute for the Ocean and Atmosphere - IPMA, Aquaculture Research Station, 1749-016, Olhão, Portugal; Portuguese Institute for the Ocean and Atmosphere - IPMA, Aquaculture Research Station, 1749-016, Olhão, Portugal; MARE - Marine and Environmental Sciences Centre, ISPA, Instituto Universitário, 1149-041, Lisbon, Portugal

**Keywords:** physiology, Diplodus sargus, developmental acclimation, climate change, Behaviour

## Abstract

Recent literature suggests that anthropogenic stressors can disrupt ecologically relevant behaviours in fish, such as the ability to escape from predators. Disruption of these behaviours at critical life history transitions, such as the transition from the pelagic environment to the juvenile/adult habitat, may have even greater repercussions. The literature suggests that an increase in temperature can affect fish escape response, as well as metabolism; however, few studies have focused on the acute sensitivity responses and the potential for acclimation through developmental plasticity. Here, we aimed at evaluating the acute and long-term effects of exposure to warming conditions on the escape response and routine metabolic rate (RMR) of early life stages of the white seabream, *Diplodus sargus*. Additionally, as food availability may modulate the response to warming, we further tested the effects of long-term exposure to high temperature and food shortage, as individual and interacting drivers, on escape response and RMR. Temperature treatments were adjusted to ambient temperature (19°C) and a high temperature (22°C). Feeding treatments were established as high ration and low ration (50% of high ration). Escape response and RMR were measured after the high temperature was reached (acute exposure) and after 4 weeks (prolonged exposure). Acute warming had a significant effect on escape response and generated an upward trend in RMR. In the long term, however, there seems to be an acclimation of the escape response and RMR. Food shortage, interacting with high temperature, led to an increase in latency response and a significant reduction in RMR. The current study provides relevant experimental data on fishes’ behavioural and physiological responses to the combined effects of multiple stressors. This knowledge can be incorporated in recruitment models, thereby contributing to fine-tuning of models required for fisheries management and species conservation.

## Teaser

Results of this study suggest an acclimation of the escape response and routine metabolic rate (RMR) of white seabream early life stages under warming conditions. Nevertheless, food shortage, interacting with high temperature, led to an increase in latency response and a significant reduction in RMRs.

## Introduction

In recent decades, the intensification of the release of carbon dioxide (CO_2_) and other polluting gases of anthropogenic origin into the atmosphere has led to profound changes in the physical and chemical properties of ocean water (Cao *et al.*, 2005; Raven *et al.*, 2005; Sabine *et al.*, 2004), with several consequences to marine life in general (Brierley and Kingsford, 2009; Domenici *et al.*, 2019; Pankhurst and Munday, 2011). Global warming has emerged as a driver of change of marine ecosystems, and fisheries in particular. Fish stocks may indirectly be affected by an increase in temperature through changes in primary and secondary productivity, which may modify distribution patterns and migratory behaviours (Brander, 2007). This will have strong implications for the fishing activity, which could suffer a reduction of 2.8–5.3% in an Representative Concentration Pathway 2.6 and 7.0–12.1% in an RCP8.5 (Barange *et al.*, 2018). Ocean warming may also impact fish species through direct effects on reproduction (Donelson *et al.*, 2010; Miller *et al.*, 2015), behaviour (Nagelkerken and Munday, 2016), physiology (Alfonso *et al.*, 2021), locomotion (Green and Fisher, 2004; Herbing, 2002) and growth (Neuheimer *et al.*, 2011; Rountrey *et al.*, 2014). However, the effects of higher temperature on marine species may be more complex than the simple relationship between individual performance and temperature (Harley *et al.*, 2006). Adding to the impacts on performance and physiology, the increase in temperature will have further effects on species interactions, such as predator–prey relationships (Allan *et al.*, 2015; Allan *et al.*, 2017; Anderson *et al.*, 2001; Bastille-Rousseau *et al.*, 2018). Avoiding predators is crucial to improve individual fitness and survival, and it has been shown that, in fish, increasing temperature can substantially impact behaviour and locomotor performance components of an escape response (Domenici *et al.*, 2019; Warren *et al.*, 2017). Increasing temperature will also increase physiological rates, which will have further effects on reproduction, growth and feeding rates (Pilakouta *et al.*, 2020; Rangel and Johnson, 2018). The routine metabolic rate (RMR), which refers to the average rate of metabolism when the animal is undergoing normal behaviours or some other specified type of activity (Metcalfe *et al.*, 2016), is one of the most used physiological characteristics to assess individual fitness. As an early response to warming, individual RMR is expected to change to compensate the increase in temperature. Yet, a prolonged exposure to these new environmental conditions might provide the organisms time to acclimate, if the cost–benefit ratio is favourable (Rummer *et al.*, 2014). However, to fully comprehend how metabolism will be affected by increasing temperature, a better understanding of organisms’ thermal sensitivity is necessary (Rangel and Johnson, 2018). Q_10_ is one of the most used thermal sensitivity measures and represents the rate of change in metabolic rates with an increase or decrease of 10°C (Rangel and Johnson, 2018). For most fish species, the mean Q_10_ value lies between 2 and 3 (Clarke and Fraser, 2004), despite being dependent on factors such as thermal regime (Donelson and Munday, 2012) and phylogeny (Clarke and Johnston, 1999).

Frequently, experimental studies addressing the effects of environmental stressors on behavioural and physiological responses of fish are held under optimal feeding conditions, which might mask the real response of individuals to the stressor (Chick and Van-Den-Avyle, 2000). In the natural habitat, where food resources are finite and not always available, the available energy may not be sufficient to support all biological functions, with some maximized in detriment of others (McMahon *et al.*, 2019). Climate change is also expected to limit food availability, due to increased ocean stratification, which will isolate the surface layers of nutrient-rich deep waters (O'Connor, 2009), potentially affecting primary production, with cascading effects to the entire trophic web (Lewandowska *et al.*, 2014; O'Connor, 2009). Thus, addressing the interacting effects of food availability and increasing temperature on species responses assumes particular relevance (Cominassi *et al.*, 2020; McLeod *et al.*, 2013), but, to date, has been poorly investigated.

Here, we aimed at evaluating the effects of higher temperature and food availability, as individual and combined stressors, on the escape response and RMR of late-stage larvae of white seabream, *Diplodus sargus*. Early life stages are a critical stage in the life cycle of fish, highly sensitive to environmental changes. These might directly and indirectly affect larval survival, with further consequences to the connectivity and replenishment of populations (Llopiz *et al.*, 2014). Predicting how survival of these early stages will change in a future ocean will thus be critical for determining ecological and economic impacts (Llopiz *et al.*, 2014). The white seabream, a highly important commercial species (Sá *et al.*, 2008), has a wide geographical distribution, being mainly abundant on the Atlantic coast and in the Mediterranean Sea (Froese and Pauly, 2019). After a dispersive larval phase in the plankton, late-stage larvae move to coastal regions, in particular the tidal zones, to settle, grow and reproduce (Macpherson and Raventos, 2006). In the Portuguese coast, settlement in coastal areas occurs mainly during the spring and summer months, when average temperatures range from 15°C to 18°C, but can reach up to 20–23°C in particularly warm years (Madeira *et al.*, 2012).

To assess the effects of elevated temperature and food availability, fish were reared in a fully crossed experimental design with two temperatures and two food levels. The temperature treatments were set to an ambient temperature (19°C), which can be found in their habitat (Madeira *et al.*, 2012), and the high-temperature scenario to +3°C, which is in line with projected future climate change conditions, according to the IPCC RCP scenario 8.5 (Lee *et al.*, 2021). Escape responses and RMR were measured immediately after high temperature was attained (acute response, Day 0 of treatment) and after a 4-week exposure period to temperature and food treatments. This approach allowed us to assess the following: (i) the acute response to temperature increase, (ii) the potential to acclimate to increasing temperature and (iii) the single and interacting effects of temperature and food levels. In view of all known effects of temperature on neural and sensory capacity, temperature is expected to have a detrimental effect on latency and directionality, but a beneficial one on responsiveness and locomotor parameters of the escape response (Domenici *et al.*, 2019; Warren *et al.*, 2017). Moreover, the RMR is expected to increase as temperature rises, by increasing biochemical reactions in the short term (Kieffer *et al.*, 2014; Strobel *et al.*, 2012), but in the long-term exposure we expect that a thermal acclimation will occur, placing RMR values close to control levels. The presence of food shortage conditions will likely amplify the negative effects of high temperature, by reducing available energy (Bignami *et al.*, 2017). Thus, we expect that most notorious effects in behaviour and metabolism will be observed when fish are exposed to the interacting effects of temperature and food shortage.

## Material and methods

### Ethics

This study was carried out under the approval of Direção-Geral de Alimentação e Veterinária (DGAV, Portuguese Authority for Animal Health, permit 0421/000/000/2020) and according to the University’s animal ethics guidelines.

### Fish rearing and experimental setup treatments

Experiments ran from 8 April to 28 May 2021. Approximately 250 white seabream, *D. sargus*, individuals, at the age of 48 days post-hatch, were provided by Olhã Aquaculture Research Station (EPPO, Portual), an Aquaculture Research Station (IPMA, Olhão, Portugal). Spawn was naturally obtained from a broodstock composed of 29 specimens of wild and F1 (first generation obtained at EPPO). Fish were transported to Instituto Universitário fish facilities and placed in an 80-l acclimation tank, enriched with artificial algae and sediment, with temperature (19°C), salinity (35 ppt) and photoperiod (14L:10D) matching the conditions found at the EPPO station. Fish were fed five times daily with commercial fish feed (SPAROS, Olhão, Portugal; with 60% crude protein, 15% crude fat, 12% crude ash), until satiation, and left in this tank for 10 days. Subsequently, fish were transferred to 30-l tanks, also enriched with algae and sediment, at a density of 25 individuals per tank (8 tanks in total), and left to acclimate to the new tanks for 3 days, at the same temperature, salinity and photoperiod conditions as previously described. Each tank had a continuous supply of recirculating seawater and was connected to a sump, equipped with mechanical, biological, chemical and ultraviolet filtration. During this period, fish were fed five times daily with the commercial fish feed, until satiation, as in the acclimation tank. Afterwards, fish were randomly assigned to one of four treatments: (A) ambient temperature (19°C) with high ration, (B) ambient temperature (19°C) with low ration, (C) increased temperature (22°C) with high ration and (D) increased temperature (22°C) with low ration. Ambient temperature was kept at 19.44 ± 0.03°C, and high temperature at 21.94 ± 0.04°C. Fish were randomly allocated to two replicate treatment tanks in order to ensure a homogeneous size distribution per tank. Temperature in treatments C and D were stepwise increased ~1°C per day, using heaters, to avoid any stress and heat shock associated with rapid temperature changes (Gardiner and Munday, 2010), until +3°C above the ambient temperature treatment was reached (i.e. temperature was increased throughout a 3-day period). Once the experimental temperature was reached, the food treatments started (Day 0 of treatments). Fish at the high-ration treatments were fed daily with 0.8 g of commercial fish feed (average, ~10% of fish body weight per day, BW d^−1^), divided by five meals, while fish in low-ration treatments were fed daily with 0.4 g (average, *~*5% of fish BW d^−1^), divided by five meals. The choice of the high ration was selected based on feeding protocols at EPPO station, where usually food is given *ad libitum* to assure that every larva is able to reach food, and also based on preliminary observations that allowed us to conclude that 0.8 g per day would match fish needs. The choice of low ration as 50% of the high ration was selected taking into consideration that even lower feeding levels would lead to higher mortality rates within the first few days, which we avoided. The amount of food in each tank was readjusted as mortality occurred. Fish were exposed to these conditions for 4 weeks. Temperature and salinity were measured daily, and other water quality parameters, such as ammonia, nitrates and nitrites, were monitored twice a week and kept below critical levels. Tanks were cleaned daily to remove excess of food and were equipped with filtrations systems, ensuring water quality throughout the experimental period.

Escape response and RMR were measured: (i) immediately after high temperature was attained (acute response, Day 0 of treatments) and (ii) after a 4-week exposure period to both temperature (19°C and 22°C) and food treatments (low and high rations). Each individual was only tested in a single assay, and individuals were randomly sourced from both replicate tanks per treatment. After each test, fish were euthanized with an excessive dose of MS222, weighed and total and standard lengths registered. Fulton condition K and specific growth rate (SGR, % d^−1^) were determined using the following formula:

$$ \begin{align*}& K=100\ast ( Weight/ Lengh{t}^3)\\
&\quad \frac{SGR=100\ x\ \left[ In\ \left( Weight\ initial\right)- In\left( Weight\ fina\right)\right]}{Number\ of\ days}\end{align*} $$

Approximately 11–21 individuals were analysed per treatment to determine escape response, and 10–12 individuals were analysed per treatment to determine RMR. Experimental trials were conducted over a period of 2–4 days in a temperature-controlled room to minimize temperature fluctuations during trials.

### Escape response

The experimental setup used to assess the escape response was based on protocols established by Warren *et al.* (2017), which involves presenting a stimulus to effectively elicit a startle response to the fish. Fish were individually transferred to a test arena, which consisted of a circular transparent acrylic enclosure (diameter, 19 cm) placed in a tank, with a water depth of 8 cm, to limit vertical movements during the escape response. Water temperature in the arena matched the temperature of the treatment of origin and was replaced every three trials (we cannot rule out the possibility that excretion of chemical signals between trials influenced the escape responses, but *ad libitum* observations during the habituation stage did not suggest altered behaviours). After a habituation period of 5–10 minutes, the startle response was elicited by dropping a weight attached to a wire so that it just touched the surface of the water and did not collide with the individual. To provide a sudden stimulus, the weight was dropped through a PVC tube to not elicit a visual stimulation before mechanical stimulation. The stimulus was dropped when the fish was found less than two body lengths away from the centre of the arena. The escape response was recorded using a high-speed video camera (Sony Cyber-Shot DSC-RX100M4) and a mirror was positioned at a 45° angle from the arena, so that the fish movement could be recorded to minimize visual disturbances. The test arena was surrounded by a covered structure (black curtains).

Escape response analysis included responsiveness (proportions of fish that responded to the stimulus with C-Start or S-Start), directionality (defined by the movement of the head away or towards the side stimulus), latency (interval between the initiation of the stimulus and the response developed by the individual), escape distance (distance travelled during the response) and maximum speed (maximum acceleration reached during escape response) and were chosen as they are considered reliable indicators of fish escape performance (Walker *et al.*, 2005). Videos were converted into frames (300 frames per second for latency analysis and 10 frames per second for maximum speed and escape distance analysis) and analysed using the ImageJ software with the manual tracking plugin.

### RMR and thermal sensitivity

A closed system respirometry was used to estimate individual RMRs through oxygen consumption in fish with a body weight between 0.041 and 1.54 g. Four 100-ml cylindrical o-ring chambers were connected, via a PVC tube, to 30-l tanks containing oxygen-saturated water. A stirring bar was used to ensure the homogenization of dissolved oxygen in the respiratory chamber. The water temperature inside these tanks was kept at 19 or 22°C, depending on the temperature of the treatment, using heaters. Temperature inside the respirometry chambers was maintained by submerging the chamber in a water-bath, controlled with heaters. The oxygen concentration in the chamber was measured every 15 seconds, using contactless oxygen sensor spots (OXSP5, Pyroscience), and monitored using the software Pyro Oxygen Logger (Pyroscience, Denmark). The oxygen concentration was maintained above 80% during experimental tests. To minimize the effects of bacterial respiration, the water was filtered using UV and the background bacterial oxygen consumption was recorded after each test. Between tests the chambers were disinfected and the oxygen sensors recalibrated.

From the oxygen consumption data, the thermal sensitivity was also analysed, through the Q_10_, using the following formula:

$$ {Q}_{10}={\left(\frac{RMR\ {22}^{\circ }C}{RMR\ {19}^{\circ }C}\right)}^{10^{\circ }C/\left({22}^{\circ }C-{19}^{\circ }C\right)} $$

Fish were fasted for 24 hours prior to analysis to ensure a post-absorptive state, as metabolic rates tend to increase during digestion (Killen *et al.*, 2014). After being transferred to the respiratory chamber, the individuals remained in acclimation for 1 hour with constant water circulation, before RMR analysis. Afterwards, the circulation of water inside the chamber was interrupted and the individuals remained inside the closed chamber for a period of 20 minutes, in which the oxygen consumption values were registered. During the experimental period, the respiratory chambers were covered with black plastic to avoid external disturbances, and opaque materials were used between chambers to avoid visual stimulation between subjects in the same trial. After the measurement, the water flow inside the chamber was opened again so that oxygen levels inside the respirometry chamber could reach 100% saturation. R^2^ values above 90% were considered, and the RMR was calculated using the following formula:

$$ \begin{align*} M{O}_2&=\frac{Chamber\ volume\ast Slope\ of\ oxygen\ consumption}{Weight\ (Kg)\ast Time\ (hours)}\\ &\quad- Background \end{align*}

### Data analysis

Latency (ms), maximum speed (converted to body length s^−1^ to control for any size effect), escape distance (cm), RMR (mg O_2_ Kg^−1^ h^−1^), Fulton K, SGR (% d^−1^), weight (g) and total length (cm) were analysed using Linear Mixed Models (LMMs). Escape distance, latency and Fulton K were log transformed. Responsiveness, directionality and mortality rate were assessed using a Generalized Linear Mixed Model (GLMM), fitted to a binomial distribution. Tukey’s HSD *post hoc* tests to examine differences between treatments.

To determine the effects of acute vs. prolonged exposure to ambient and high temperature on response variables, mixed models (LMM or GLMM) were used with the interaction temperature*timepoint as fixed factor, and holding tank included as a random factor. To determine the effects of temperature and food availability on response variables, mixed models (LMM or GLMM) were used with temperature and food level as fixed factors, and holding tank included as a random factor. Model assumptions were checked using the package DHARMa (Harting, 2021). Values are reported as means ± standard error (S.E.), and *P*-values below 0.05 were considered significant.

Statistical analyses were performed with R statistical software, using ‘lme4’ and ‘nlme’ package for running GLM models (R Core Team, 2018).

## Results

### Temperature effects on growth, body condition, behaviour and metabolism

During the 4-week experimental period, seabream’s total length and weight increased from 2.18 ± 0.09 cm to 3.64 ± 0.11 cm and 0.14 ± 0.01 g to 0.79 ± 0.06 g, respectively, in the ambient temperature and from 2.21 ± 0.09 cm to 3.68 ± 0.08 cm and 0.21 ± 0.04 g to 0.77 ± 0.05 g, respectively, in the high-temperature treatment. Exposure to high temperature for a 4-week period did not induce changes in white seabream Fulton’s K condition, neither on SGR, which ranged from 2.13 ± 0.23% d^−1^ at 19°C to 1.77 ± 0.29% d^−1^ (Table S1).

An acute or prolonged exposure to high temperature did not induce significant changes in the non-locomotor parameters of the escape response—responsiveness, directionality and latency. After an acute exposure to 22°C, 80% of individuals responded to stimuli with a C-Start or S-Start, while at ambient temperature the percentage of responsive individuals was higher, 95% (Table S1). After 4 weeks of exposure, responsiveness is reduced by 5% in both treatments (Table S2). Like responsiveness, directionality did not differ between temperature treatments after an acute or prolonged exposure, with all fish moving in the opposite direction of the stimulus. At ambient temperature, latency to respond to the stimulus did not change with exposure time, averaging 12.61 ± 3.06 ms; at higher temperature, latency doubled, increasing from 16.50 ± 4.11 ms to 32.21 ± 11.31 ms after 4 weeks of exposure (not significantly; Table S1).

**Figure 1:**
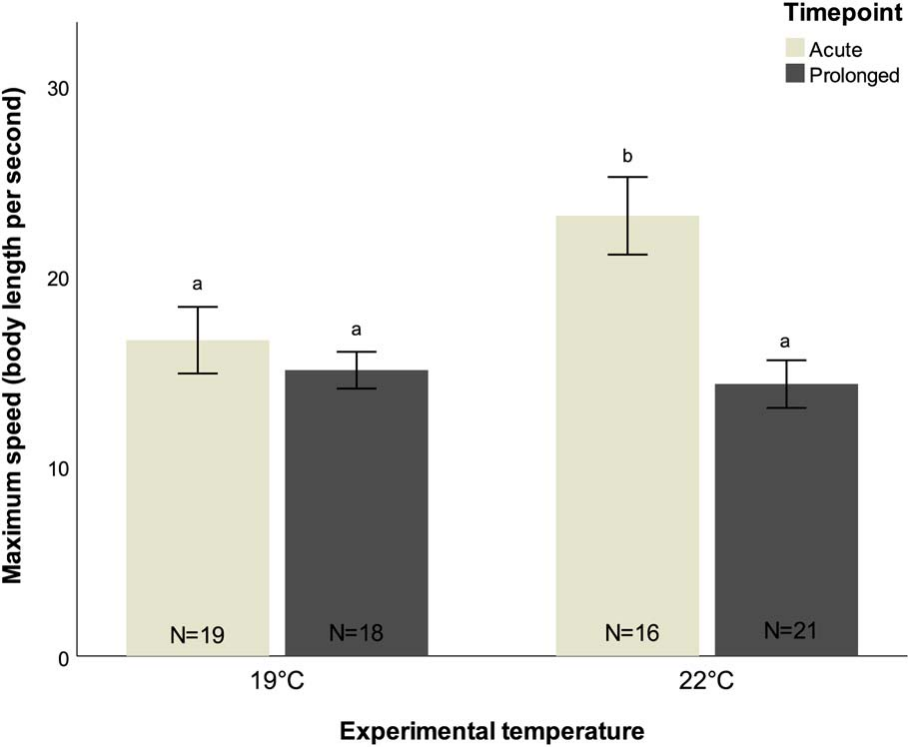
Maximum speed (body length per second) of *D. sargus* reared under ambient and high-temperature treatments, following an acute and prolonged exposure. Letters represent significant differences between treatments. Values are reported as mean ± S.E.

Temperature had a significant effect on the maximum speed attained during the escape response. At 19°C, a maximum acceleration of 16.61 ± 1.75 BL s^−1^ was recorded, which increased to 23.15 ± 2.04 BL s^−1^ at 22°C, corresponding to a 40% increase (*t*-value = 2.949, *P* = 0.004; Fig. 1). At ambient temperature, maximum speed did not change with exposure time, but interestingly, after a prolonged exposure to 22°C, maximum speed reduced significantly, from 23.15 ± 2.04 BL s^−1^ to 14.30 ± 1.22 BL s^−1^ (Tukey test, *z*-value = −4.080, *P* < 0.001), which is similar to maximum speed values attained by fish kept at ambient temperature (Table S2). After an acute exposure to 22°C, fish covered twice the distance as a response to the stimulus compared with fish under ambient temperature (6.89 ± 0.72 vs 14.09 ± 3.43 cm), but after a prolonged exposure escape distances were similar to the ones covered by fish kept at ambient temperature (ambient, 9.12 ± 0.92 cm; high, 11.35 ± 1.39 cm) (Table S1).

**Figure 2:**
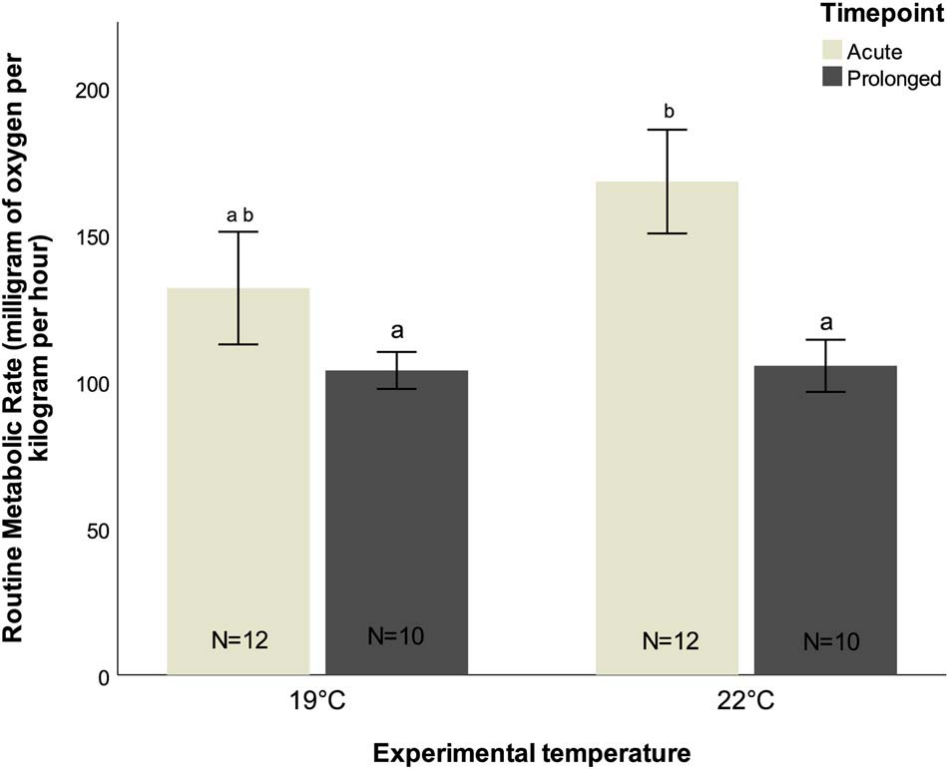
RMR (milligram of oxygen per kilogramme per hour) of *D. sargus* reared under ambient and high-temperature treatments, following an acute and prolonged exposure. Letters represent significant differences between treatments. Values are reported as mean ± S.E.

Regarding metabolism (Fig. 2; Tables S1, S2), an acute exposure to 22°C led to an increase of RMR of ~13%, although not significantly, and a prolonged exposure to this temperature was not reflected in changes in oxygen consumption either, in comparison to fish kept under ambient temperature for the same period of time (Tables S1, S2). The RMR slightly decreased between timepoints at ambient temperature, although not significantly, while at 22°C there was a significant reduction of more than 37% in oxygen consumption after a 4-week exposure (Tukey test, *z*-value = −2.924, *P* = 0.018) (Tables S1, S2). With acute exposure to elevated temperature, a Q_10_ of 2.25 was recorded, which decreased after 4 weeks of exposure to 1.05.

### Combined effects of temperature and food availability on mortality, body condition, behaviour and metabolism

Fulton’s K condition did not significantly differ between fish reared under the different food and temperature treatments, neither did body weight, with values ranging between 0.70 ± 0.06 and 0.79 ± 0.06 g, or total length, with values ranging from 3.47 ± 0.11 to 3.72 ± 0.10 cm (Table S3). At 19°C, a mortality rate of 6% was recorded in the high-ration treatment after a 4-week exposure, which increased to 9.80% with the reduction of food availability (Table S3). At 22°C, mortality rate increased from 2% in the high-ration treatment to 18% in the low-ration treatment (Table S3).

**Figure 3:**
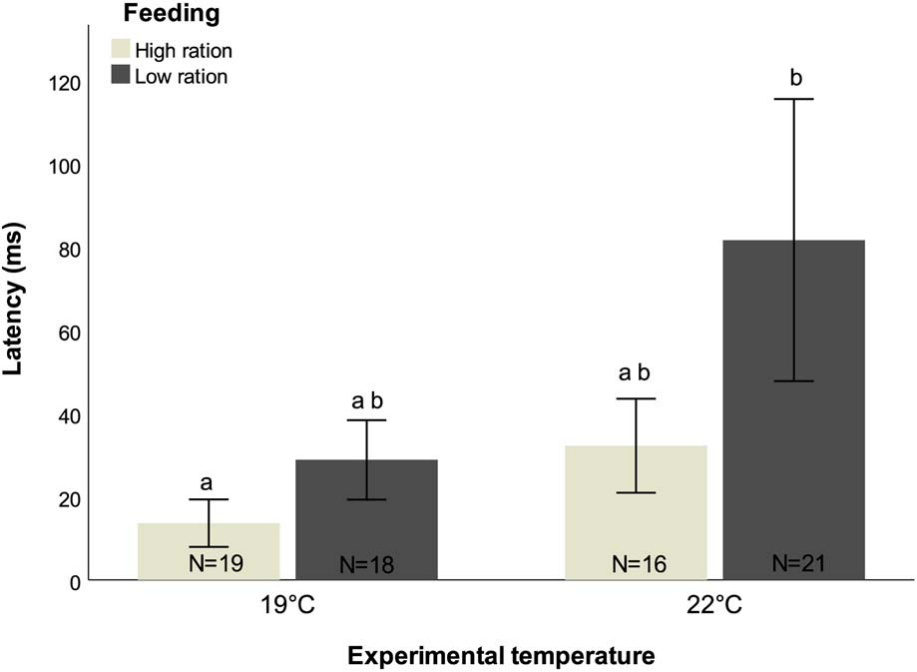
Latency (millisecond) of *D. sargus* reared under high-ration and low-ration treatments, at ambient and high temperature, following prolonged exposure. Letters represent significant differences between treatments. Values are reported as mean ± S.E.

At both ambient and high temperatures, responsiveness did not change with feeding regime, ranging from 90% to 94.74% at 19°C and 75% to 78.57% at 22°C (Table S3). Likewise, temperature did not affect responsiveness between feeding regimes (Table S3). Similarly, directionality did not differ between feeding treatments in both temperatures, with more than 90% of all individuals across all treatments responding in the opposite direction to the stimulus. At ambient temperature, food availability did not significantly affect latency response, although there was a tendency to increase with food shortage, from 13.63 ± 5.70 to 28.82 ± 9.56 ms (Fig. 3; Tables S3, S4). At 22°C, a similar trend was observed. When well fed (high ration), latency did not differ between ambient and high temperatures; however, the combination of high temperature and low ration significantly increased latency response (*t*-value = 2.638, *P* = 0.010) (Fig. 3; Tables S3, S4).

Maximum speed attained in the escape response was not affected by food shortage, in neither temperature treatments, ranging from 14.64 ± 1.34 to 15.03 ± 0.97 BL s^−1^ at 19°C and from 11.89 ± 1.31 to 14.30 ± 1.12 BL s^−1^ at 22°C (Table S3). The combination of high temperature and low ration led to a small decrease of the maximum speed (20.93%), although not significantly (Table S3). Likewise, different feeding regimes did not affect the escape distance covered by fish reared at ambient and high temperatures (Table S3). Nevertheless, and as seen for maximum speed, the interaction of high temperature and low ration lead to a decrease in the covered distance compared with fish reared under control temperature and high ration, but not significantly (−1.38 cm, 95% CI: [0.71–1.03]) (Table S3).

**Figure 4:**
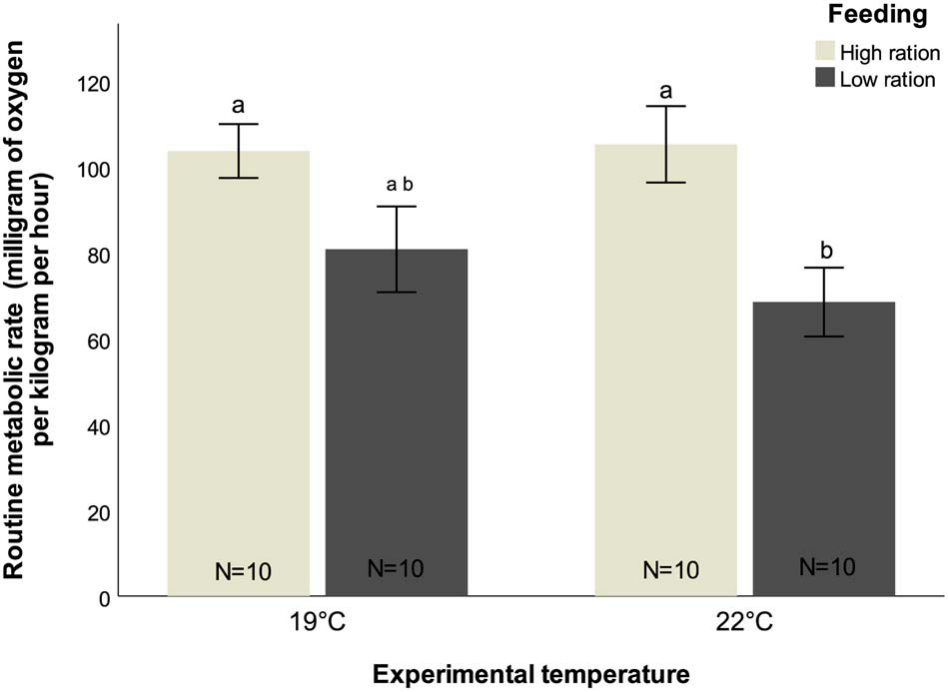
RMR (milligram of oxygen per kilogramme per hour) of *D. sargus* reared under high-ration and low-ration treatments, at ambient and high temperature, following prolonged exposure. Letters represent significant differences between treatments. Values are reported as mean ± S.E.

As for metabolism, food availability together with temperature had a significant effect on RMR (*t*-value = 2.568, *P* = 0.014) (Fig. 4; Tables S3 , S4). RMR decreased between feeding conditions at 19°C, from 103.65 ± 6.29 to 80.78 ± 9.98 mg O_2_ Kg^−1^ h^−1^, although this decrease is marginally non-significant. With exposure to 22°C, significant differences were observed between feeding regimes (Tukey test, *z*-value = −2.560, *P* = 0.050), with RMR decreasing from 105.21 ± 8.89 mg O_2_ Kg^−1^ h^−1^ in the high-ration conditions to 68.47 ± 8.02 mg O_2_ Kg^−1^ h^−1^ under low-ration conditions. RMR did not differ significantly between temperature treatments in the high-ration and low-ration conditions (Fig. 4; Tables S3, S4).

## Discussion

### Temperature effects on growth, body condition, behaviour and metabolism

Evaluating how temperature affects the escape response of marine species is essential to understand how predator–prey interactions will be affected by climate change (Gilman *et al.*, 2010). Here, we observed that a rapid increase in temperature caused changes in the locomotor parameters of the escape response—distance and escape velocity—of white seabream, but not in the non-locomotor elements—responsiveness, directionality and latency. Changes in locomotor parameters with temperature must be associated with an increase in the muscle shortening speed, which contributes to an increase in muscle power and, consequently, a greater locomotor performance (Wakeling *et al.*, 2000), which corroborates the higher maximum speed and escape distance observed in the current study after a rapid increase in temperature. These results contradict those obtained by Allan *et al.* (2017) in the tropical ward’s damselfish (*Pomacentrus wardi*), in which the authors found a tendency to reduce the maximum speed (from values close to 0.45–0.3 m s^−1^) and the escape distance (from 30 to 20 cm) with increasing warming. The authors argue that the decrease in maximum speed and escape distance is related to the rapid increase in standard metabolic rate and maintenance, or slow increase in maximum metabolic rate, which in turn will reduce the aerobic scope and, consequently, decrease the energy available for all processes that are not related to homeostasis, which include the locomotor parameters of the escape response (Domenici *et al.*, 2019) In fact, several studies have shown increases in metabolic rates, namely RMR, with an acute increase in temperature. Kieffer *et al.* (2014), for example, reported a 75% increase in RMR in shortnose sturgeon (*Acipenser brevirostrum*) acclimated to 10°C and subjected to an acute increase of 5°C. Strobel *et al.* (2012) even found a 101% increase in RMR with an acute temperature increase of 6°C in marbled rockcod (*Notothenia rossii*). This was not the case in our study, though, where no differences in RMR were observed after an acute exposure to +3°C. Despite the absence of a temperature effect on metabolism, our data still suggests that early life stages of white seabream are sensitive to a rapid temperature increase, as evidenced by the Q_10_ value (2.25). This value is in line with the Q_10_ values obtained in this species (2.07) (Kemp, 2009), but also in other species that also use the intertidal regions, such as the goby *Caffrogobius caffer* (1.82) (Kemp, 2009), the giant goby (*Gobius cobitis*) (2.10) (Berschick *et al.*, 1987) and the rockpool blenny (*Hypsoblennius gilberti*) (2.30) (Graham, 1970). Nevertheless, the effects of acute temperature increase on metabolism were less significant compared with tropical species, which Q_10_ values range between 4.2 and 5.7 (Nilsson *et al.*, 2009).

Contrary to what has been described in the literature, the responsiveness, directionality and latency did not differ with temperature. Szabo *et al.* (2008) and Warren *et al.* (2017) described that the increase in temperature will generate a greater responsiveness due to changes in the balance between excitatory and inhibitory synaptic transmission. However, the observed tendency for a reduction in responsiveness may suggest a decrease in overall activity, as proposed by Yocom and Edsall (1974) and Webb and Zhang (1994).

Interestingly, the results of the present study suggest that white seabream will be able to fully compensate the acute thermal effects on locomotor parameters after prolonged exposure to 22°C. After 4 weeks of exposure, the maximum acceleration and escape distance approached the values obtained at ambient temperature, suggesting the existence of a thermal acclimation, which has been described in the literature as well. Johnson and Bennett (1995) demonstrated that goldfish (*Carassius auratus*) can compensate the effects of high-temperature exposure on escape velocity after 4 weeks of exposure. Similar results were observed in golden grey mullet (*Chelon auratus*) and trinidad guppies (*Poecilia reticulata*), in which it was found that the escape velocity did not differ between fish acclimated to different temperatures, indicating the existence of full compensation after 30–70 days of exposure (Killen *et al.*, 2015; Muñoz *et al.*, 2012). Nevertheless, in the literature we also find signs of lack of acclimation. For example, in the common carp (*Cyprinus carpio*), after 2 weeks of exposure to high temperatures, changes in muscle fibre levels were induced, and thermal acclimation was not achieved (Wakeling *et al.*, 2000). The Q_10_ value also supports the thermal acclimation hypothesis of white seabream at elevated temperatures. After 4 weeks of exposure, a Q_10_ value of 1.05 was obtained, which being close to 1 suggests an independence between metabolic rates and temperature (Farrell, 2011; Seebacher *et al.*, 2015).

Thermal acclimation, despite all the costs involved, namely the lower investment in other activities, will allow the conservation of fitness, which is crucial for adapting to a changing environment (Angilletta *et al.*, 2003; Donelson *et al.*, 2011). However, this regulation will only be possible if the temperature change occurs within the thermal range of the species (Evans, 1990). Most species have a thermal range, in which a slight increase in temperature may be beneficial to the individual through greater energy supply, greater number of substrate-enzyme complexes and higher rates of diffusion (Takasuka and Aoki, 2006). However, if the increase in temperature exceeds higher pejus temperatures, body condition may be negatively affected by the existence of a cardiac output (McKenzie *et al.*, 2016; Neuheimer *et al.*, 2011), mitochondrial activity (Abele *et al.*, 2002; Lefevre *et al.*, 2021) and oxygen-carrying capacity, unable to ensure metabolic rates (Mac Millan, 2019). In the current study, we did not observe differences in seabream’s body condition (Fulton condition K and SGR) between temperature conditions. However, and considering that an increase in temperature that falls within the species thermal range would contribute to an increase in growth rates (Brander, 1995; Green and Fisher, 2004), we were expecting to have larger fish at 22°C. This lack of differences may then suggest that the costs associated with establishing acclimation may be involving a lower energy investment in body growth (Angilletta, 2009; Donelson *et al.*, 2011).

### Combined effects of temperature and food availability on mortality, body condition, behaviour and metabolism

Reducing food availability did not affect fish escape response within each temperature treatment; however, it significantly decreased RMR at 22°C. This shows that the escape response is maintained even if metabolic changes related to food limitation are taking place (Domenici and Blake, 1997; Yan *et al.*, 2015). This maintenance, namely of locomotor parameters, may indicate the absence of differences, between feeding conditions, in the concentration of ATP, phosphocreatine and glycogen, which are the main energy sources used during the escape response (Kieffer *et al.*, 1996; Kieffer, 2000; Downie *et al.*, 2020). Thus, the results obtained suggest that white seabream, subject to reduced food availability, do not exercise energy conservation and have the ability to preserve the anaerobic capacity, as a way to ensure the escape of predators in famine circumstances (Gingerich *et al.*, 2010; Skajaaa and Browman, 2007). However, it must also be hypothesized that a 50% reduction in food availability may not have been enough to change the condition and behaviour of this species. Although food shortage did not induce changes in the escape response, by itself, the interaction of high temperature and reduced food availability significantly increased latency compared with the control treatment (19°C + high ration). We discuss this result in the light of the energy-saving hypothesis, which suggests that under stressful conditions, individuals can model their response, namely by increasing latency, if they do not consider the stimulus as a high-threat risk factor, in order to conserve energy (Ramasamy *et al.*, 2015). In the current study, the stimulus intensity may not have been considered a high-threat factor.

The reduction in food availability led to a decreasing trend (22%) of the RMR at 19°C. These results are less expressive than those obtained in species of the Cyprinidae family, which show a 40% reduction in RMR in individuals kept under food restrictions conditions (Wieser *et al.*, 1992); in the rainbow trout (*Oncorhynchus mykiss*) and in California scorpionfish and shortspine thornyhead, in which 60% and 66% reductions in routine oxygen consumption were reported, after 90–115 days of exposure, respectively (Yang and Somero, 1993). The interaction of food limitation and high temperature had a significant effect on RMR, with almost 35% reduction compared with fish fed with high ration at 19°C. These results are in line with those obtained in juveniles of European perch (*Perca fluviatilis*), in which there was a 57% reduction in routine oxygen consumption after 14 days of exposure to high temperatures and food restrictions (Mehner and Wieser, 1994). This suggests that fish regulate their metabolic rates depending on the food availability and/or temperature, to minimize the effects on body condition (O'Connor *et al.*, 2000). The difference in RMR effects between species can be explained by more or less significant effects of food availability on the biochemical composition of tissues, on the reduction of the relative size of certain organs and on the synthesis and turnover of proteins and cellular components (O'Connor *et al.*, 2000). This decrease in the volume of certain organs and protein synthesis may also contribute to the loss of physical condition.

Contrary to our expectations, total length and weight did not differ between the two feeding conditions. It is likely that these two variables may have been significantly reduced soon after the introduction of food restrictions, as described by Ziegelbecker and Sefc (2021). But later, size and weight increased to values close to high-ration treatment, suggesting that white seabream invested in body condition, perhaps through a reduction of activity levels, which is consistent with the need to reduce risks of predation (Ziegelbecker and Sefc, 2021). Unfortunately, we did not assess fish activity levels under the different treatments; therefore, we can only speculate that individuals under food deprivation and high temperature were less active as an energy-saving strategy. Prolonged exposure to food restrictions may also have allowed individuals to adapt to these conditions, through greater digestive efficiency (Kotrschal *et al.*, 2014) or adjustments in metabolic rates (O'Connor *et al.*, 2000). However, it is necessary to hypothesize that there may have been a size-dependent mortality that prevented us from finding significant differences between treatments and that a 50% reduction in food availability could still be within the limits supported by the species, without causing effects on body condition. The choice of the low ration level in the current study was quite conservative as we are handling fish in a stage of rapid growth and high energetic demands and we did not want to induce high mortality due to starvation. In the wild, though, food is rarely unlimited and even a feeding level of 5% BW d^−1^ might be considered a high food level. Our results can, therefore, be considered conservative and the combination of higher temperature and food deprivation in the wild might lead to more meaningful responses than the ones observed here.

Mortality was higher in low-ration treatments, ranging between 10% and 18%. This indicates that food availability plays a more important role in survival than temperature, but the combined effect of these two drivers increased mortality rate. Exposure to low food availability, in addition to affecting the synthesis of cellular components and internal organ volume, will contribute to the atrophy of adipose tissues and reduced immune capacity, which will have an impact on growth and survival in the early stages of development (Park *et al.*, 2012). This reduction in immune capacity will be particularly relevant given the increased development of pathogens at high temperatures (Martins *et al.*, 2011A). However, some studies propose that the increase in temperature may promote a greater antibody response (Martins *et al.*, 2011b) and an innate immunity via behavioural fever, generating higher survival rates (Boltaña *et al.*, 2013). The interaction of reduced food availability with increasing temperature on RMR might further contribute to increased oxidative stress and mortality, through lactic acidosis and heart failure (Bignami *et al.*, 2017; Farrel, 2009).

## Conclusion

The main objectives of this study were to (i) evaluate the acute response to temperature increase; (ii) analyse the potential for acclimation to increased temperature; and (iii) evaluate the combined effect of temperature and food availability, as individual and interacting factors, on escape response and RMR. The results obtained show that the acute warming increases the locomotor parameters of the escape response, but does not significantly affect the non-locomotor parameters (responsiveness and directionality) and RMR. This is particularly relevant because much of the habitat of this species (Atlantic and Mediterranean Sea) is subject to the effects of marine heat waves, with greater frequency in the Atlantic, but of greater intensity and duration in the Mediterranean (Galli *et al.*, 2017; Plecha *et al.*, 2021). The duration, intensity and frequency of these events will be intensified by advancing climate change (Galli *et al.*, 2017). After prolonged exposure to 22°C, the locomotor parameters approach the values obtained at ambient temperature, suggesting the existence of an acclimation process. Fulton’s K condition, body weight and total length did not differ between different temperature conditions and food availability. Also, the escape response is maintained under conditions of reduced food availability, at both ambient and high temperatures. However, the interaction of these stressors significantly increased latency and reduced RMR.

Altogether, this study suggests that early life stages of white seabream may be able to conserve their ability to escape predators, but will undergo metabolic changes with variations in temperature and food availability. We should further stress that results here obtained are quite conservative, as the temperature level selected as high (22°C) is still within the thermal regime that the species may experience in the natural habitat. Temperature was a driver for biological changes in our case, but future extreme temperature conditions might actually act as a true stressor, inducing much severe and negative consequence for fish (Boyd and Hutchins, 2012). Therefore, it would be important for future experiments to include greater thermal amplitude. It would also be important to evaluate the escape response in white seabream as a group, considering that it is a species that lives in schools (Froese and Pauly, 2019). In addition, it would be relevant to determine how predators will respond to climate change in terms of predator–prey interactions (capture rates, attack rates and maximum speed reached during attack) and metabolism and develop a broader multi-stress analysis (Domenici *et al.*, 2019).

The behavioural and physiological data here obtained can contribute to fine-tune species recruitment models, providing a better understanding of how climate change will affect species responses, as these will differ depending upon whether the stressors work in an additive, synergistic or antagonistic way on the various levels of biological organization. Integrating both experimental and ecological data into recruitment models will provide a solid basis on which stakeholders decisions can be supported.

## Funding

The authors acknowledge the European Regional Development Fund (FEDER) through the Lisbon's Regional Operational Programme (LISBOA-01-0145-FEDER-031532) and the Portuguese Foundation for Science & Technology (FCT) through the project NextGen (PTDC/CTA-AMB/31532/2017) and the strategic project MARE-UIDB/04292/2020 granted to MARE (Marine and Environmental Sciences Centre). The authors also acknowledge DiversiaquaII (Mar2020-P02M01-0656P).

## Author contributions

A.M.F. designed the study. J.A. and A.R.L. performed the experimental work. L.R. contributed to the experimental design and editing of the manuscript. S.C., A.M. and P.P.F. provided the fish at the age of 48 dph. J.A., A.R.L. and A.M.F. performed data analysis and wrote the manuscript.

## Data availability

Data available upon request from the authors.

## Supplementary Material

supplementary_coac023
