# Correction to: Effects of exposure to elevated temperature and different food levels on the escape response and metabolism of early life stages of white seabream, Diplodus sargus

**DOI:** 10.1093/conphys/coad047

**Published:** 2023-06-28

*Conserv Physiol* 10(1): coad047; doi: 10.1093/conphys/coad047

In the originally published version of this manuscript, the Funding section was given as follows:

This work was supported by the strategic Project MARE/UIDB/MAR/04292/2020 and the Project NextGen (PTDC/CTAAMB/31532/2017), co-financed by Science and Technology Foundation through national funds, and by the European Union through FEDER (European Regional Development Fund) and Diversiaqua II (Mar2020-P02M01-0656P).

The Funding section has been corrected to read as follows:

The authors acknowledge the European Regional Development Fund (FEDER) through the Lisbon's Regional Operational Programme (LISBOA-01-0145-FEDER-031532) and the Portuguese Foundation for Science & Technology (FCT) through the project NextGen (PTDC/CTA-AMB/31532/2017) and the strategic project MARE-UIDB/04292/2020 granted to MARE (Marine and Environmental Sciences Centre). The authors also acknowledge DiversiaquaII (Mar2020-P02M01-0656P).

