# Supplementary material for: Effects of exposure to elevated temperature and different food levels on the escape response and metabolism of early life stages of white seabream, Diplodus sargus
Source: Conserv Physiol. 2022 May 6;10(1):coac023. doi: 10.1093/conphys/coac023 (PMC9109722; doi:10.1093/conphys/coac023)
Supplement: supplementary_coac023 [file supplementary_coac023.docx]

**Supplementary materials for:**

**Effects of exposure to elevated temperature and different food levels on the escape response and metabolism of early life stages of white seabream, *Diplodus sargus***

João Almeida^1^, Ana Rita Lopes^1,2^, Laura Ribeiro^3^, Sara Castanho^3^, Ana Candeias- Mendes^3^, Pedro Pousão-Ferreira^3^, Ana M. Faria^1,*^

^1.^ MARE - Marine and Environmental Sciences Centre, ISPA, Instituto Universitário, Lisbon, Portugal

^2.^ MARE - Marine and Environmental Sciences Centre, Faculdade de Ciências da Universidade de Lisboa, Lisbon, Portugal

^3.^ Portuguese Institute for the Ocean and Atmosphere - IPMA, Aquaculture Research Station, Olhão, Portugal

*corresponding author: MARE - Marine and Environmental Sciences Centre, ISPA, Instituto Universitário, Lisbon, Portugal. Tel: + 351 218 811 700. Email: afaria@ispa.pt

**Table S1 -** Statistical results of the Linear mixed effects models on Fulton’s K condition, growth rate, escape response variables and metabolism of *Diplodus sargus*. Fish were reared in two temperature treatments - ambient (19 ºC) or high temperature (22 ºC) - and tested at two timepoints - acute and prolonged (4-weeks). Shown are the parameter estimates, standard error (SE), degrees of freedom (df), t-statistic value, and P-value. Significant differences (p˂0.05) are indicated in bold.

|  | Estimate | SE | df | t value | P |
| --- | --- | --- | --- | --- | --- |
| **Fulton K** |  |  |  |  |  |
| Intercept | 0.113 | 0.034 | 4.05 | 3.344 | **0.028** |
| Temperature | 0.051 | 0.047 | 6.51 | 1.084 | 0.317 |
| Timepoint | 0.089 | 0.049 | 3.56 | 1.799 | 0.155 |
| Temperature*Timepoint | -0.091 | 0.073 | 4.089 | -1.252 | 0.277 |
|  |  |  |  |  |  |
| **Growth Rate** |  |  |  |  |  |
| Intercept | 5.789 | 0.573 | 32 | 10.100 | **< 0.0001** |
| Temperature | -1.313 | 0.836 | 32 | -1.570 | 0.126 |
|  |  |  |  |  |  |
| **Latency** |  |  |  |  |  |
| Intercept | 0.868 | 0.119 | 70 | 7.264 | **< 0.0001** |
| Temperature | 0.083 | 0.177 | 70 | 0.467 | 0.642 |
| Timepoint | -0.082 | 0.171 | 70 | -0.481 | 0.632 |
| Temperature*Timepoint | 0.199 | 0.243 | 70 | 0.818 | 0.416 |
|  |  |  |  |  |  |
| **Maximum speed** |  |  |  |  |  |
| Intercept | 16.609 | 1.500 | 70 | 11.072 | **< 0.0001** |
| Temperature | 6.543 | 2.219 | 70 | 2.949 | **0.004** |
| Timepoint | -1.577 | 2.151 | 70 | -0.733 | 0.466 |
| Temperature*Timepoint | -7.276 | 3.055 | 70 | -2.382 | **0.019** |
|  |  |  |  |  |  |
| **Distance** |  |  |  |  |  |
| Intercept | 0.811 | 0.0886 | 4.18 | 9.474 | **< 0.0001** |
| Temperature | 0.195 | 0.116 | 8.08 | 1.680 | 0.131 |
| Timepoint | 0.108 | 0.127 | 3.55 | 0.856 | 0.446 |
| Temperature*Timepoint | -0.079 | 0.184 | 4.80 | -0.429 | 0.686 |
|  |  |  |  |  |  |
| **SMR** |  |  |  |  |  |
| Intercept | 131.65 | 14.46 | 40 | 9.103 | **< 0.0001** |
| Temperature | 36.29 | 20.45 | 40 | 1.774 | **0.0536** |
| Timepoint | -28.00 | 21.45 | 40 | -1.305 | 0.199 |
| Temperature*Timepoint | -34.73 | 30.34 | 40 | -1.145 | 0.259 |
|  |  |  |  |  |  |
|  | **Estimate** | **SE** |  | **z- value** | **Pr (>\|z\|)** |
| **Responsiveness** |  |  |  |  |  |
| Intercept | 2.944 | 1.026 |  | 2.870 | **0.004** |
| Temperature | -1.558 | 1.168 |  | -1.334 | 0.182 |
| Timepoint | -0.747 | 1.268 |  | -0.589 | 0.556 |
| Temperature*Timepoint | 0.459 | 1.453 |  | 0.316 | 0.752 |

**Table S2 –** Tukey tests on the maximum speed and SMR of Diplodus sargus. Fish were reared in two temperature treatments - ambient (19 ºC) or high temperature (22 ºC) - and tested at two timepoints - acute and prolonged (4-weeks). Shown are the parameter estimates, standard error (SE), z-value, and Pr value. Significant differences (p˂0.05) are indicated in bold.

|  | **Estimate** | **SE** | **z-value** | **Pr (>\|z\|)** |
| --- | --- | --- | --- | --- |
| **Maximum speed** |  |  |  |  |
| 22ºC x acute – 19 ºC x acute | 6.542 | 2.219 | 2.949 | **0.0170** |
| 19ºC x prolonged - 19 ºC x acute | -1.577 | 2.151 | -0.733 | 0.884 |
| 22ºC x prolonged - 19 ºC x acute | -2.310 | 2.070 | -1.116 | 0.679 |
| 19ºC x prolonged – 22ºC x acute | -8.120 | 2.247 | -3.614 | **0.002** |
| 22ºC x prolonged – 22ºC x acute | -8.853 | 2.169 | -4.080 | **<0.001** |
| 22ºC x prolonged – 19ºC x prolonged | -0.733 | 2.100 | -0.349 | 0.985 |
|  |  |  |  |  |
| **Metabolism** |  |  |  |  |
| 22ºC x acute – 19 ºC x acute | 36.291 | 20.453 | 1.774 | 0.285 |
| 19ºC x prolonged - 19 ºC x acute | -28.001 | 21.451 | -1.305 | 0.559 |
| 22ºC x prolonged - 19 ºC x acute | -26.436 | 21.451 | -1.232 | 0.606 |
| 19ºC x prolonged – 22ºC x acute | -64.292 | 21.451 | -2.997 | **0.015** |
| 22ºC x prolonged – 22ºC x acute | -62.727 | 21.451 | -2.924 | **0.018** |
| 22ºC x prolonged – 19ºC x prolonged | 1.565 | 22.405 | 0.070 | 0.999 |

**Table S3 -** Statistical results of the Linear mixed effects models on the Fulton’s K condition, body weight, total length, escape response variables, metabolism and mortality rate of Diplodus sargus. Fish were reared in either high or low food treatments, cross factored with ambient (19 ºC) or high temperature (22 ºC). Shown are the parameter estimates, standard error (SE), degrees of freedom (df), t-statistic value, and P-value. Significant differences (p˂0.05) are indicated in bold. A: high ration x ambient temperature; B: high ration x high temperature; C: low ration x ambient temperature; and D: low ration x high temperature.

|  | Estimate | SE | df | t value | P |
| --- | --- | --- | --- | --- | --- |
| **Fulton K** |  |  |  |  |  |
| Intercept | 0.202 | 0.025 | 2.76 | 8.192 | **0.005** |
| Treatment B | -0.012 | 0.034 | 2.76 | -0.328 | 0.766 |
| Treatment C | -0.001 | 0.035 | 2.73 | -0.024 | 0.982 |
| Treatment D | -0.029 | 0.038 | 3.19 | -0.772 | 0.493 |
|  |  |  |  |  |  |
| **Body weight** |  |  |  |  |  |
| Intercept | 0.782 | 0.064 | 64 | 12.273 | **< 0.0001** |
| Treatment B | -0.014 | 0.087 | 64 | -0.158 | 0.875 |
| Treatment C | -0.097 | 0.090 | 64 | -1.072 | 0.288 |
| Treatment D | -0.024 | 0.103 | 64 | -0.235 | 0.815 |
|  |  |  |  |  |  |
| **Total length** |  |  |  |  |  |
| Intercept | 3.611 | 0.110 | 64 | 32.713 | **< 0.0001** |
| Treatment B | 0.056 | 0.150 | 64 | 0.369 | 0.713 |
| Treatment C | -0.156 | 0.156 | 64 | -0.996 | 0.323 |
| Treatment D | 0.080 | 0.179 | 64 | 0.445 | 0.658 |
|  |  |  |  |  |  |
| **Latency** |  |  |  |  |  |
| Intercept | 0.786 | 0.148 | 64 | 5.311 | **< 0.0001** |
| Treatment B | 0.282 | 0.202 | 64 | 1.397 | 0.167 |
| Treatment C | 0.223 | 0.209 | 64 | 1.064 | 0.291 |
| Treatment D | 0.634 | 0.240 | 64 | 2.638 | **0.010** |
|  |  |  |  |  |  |
| **Maximum speed** |  |  |  |  |  |
| Intercept | 14.670 | 2.213 | 2.938 | 6.63 | **0.007** |
| Treatment B | -1.011 | 3.176 | 2.93 | -0.318 | 0.772 |
| Treatment C | -0.759 | 3.113 | 2.91 | -0.244 | 0.823 |
| Treatment D | -3.729 | 3.451 | 3.38 | -1.081 | 0.351 |
|  |  |  |  |  |  |
| **Distance** |  |  |  |  |  |
| Intercept | 0.9233 | 0.053 | 64 | 17.476 | **< 0.0001** |
| Treatment B | 0.0752 | 0.072 | 64 | 1.045 | 0.300 |
| Treatment C | 0.0194 | 0.074 | 64 | 0.260 | 0.796 |
| Treatment D | -0.075 | 0.086 | 64 | -0.876 | 0.384 |
|  |  |  |  |  |  |
| **SMR** |  |  |  |  |  |
| Intercept | 2.008 | 0.056 | 36 | 35.700 | **< 0.0001** |
| Treatment B | -0.001 | 0.079 | 36 | -0.008 | 0.994 |
| Treatment C | -0.143 | 0.079 | 36 | -1.796 | 0.081 |
| Treatment D | -0.204 | 0.079 | 36 | -2.568 | **0.014** |
|  |  |  |  |  |  |
|  | **Estimate** | **SE** |  | **z- value** | **Pr (>\|z\|)** |
| **Responsiveness** |  |  |  |  |  |
| Intercept | 2.197 | 0.745 |  | 2.948 | **0.003** |
| Treatment B | -1.099 | 0.864 |  | -1.272 | 0.203 |
| Treatment C | 0.693 | 1.269 |  | 0.546 | 0.585 |
| Treatment D | -0.898 | 0.989 |  | -0.907 | 0.364 |
|  |  |  |  |  |  |
| **Mortality** |  |  |  |  |  |
| Intercept | 2.772 | 0623 |  | 4.448 | **< 0.0001** |
| Treatment B | 1.143 | 1.195 |  | 0.957 | 0.339 |
| Treatment C | -0.534 | 0.793 |  | -0.674 | 0.500 |
| Treatment D | -1.239 | 0.736 |  | -1.682 | 0.093 |

**Table S4 –** Tukey tests on the latency response and SMR of Diplodus sargus. Fish were reared in either high or low food treatments, cross factored with ambient (19 ºC) or high temperature (22 ºC). Shown are the parameter estimates, standard error (SE), degrees of freedom (df), t-statistic value, and P-value. Significant differences (p˂0.05) are indicated in bold. A: high ration x ambient temperature; B: high ration x high temperature; C: low ration x ambient temperature; and D: low ration x high temperature.

|  | **Estimate** | **SE** | **z-value** | **Pr (>\|z\|)** |
| --- | --- | --- | --- | --- |
| **Latency** |  |  |  |  |
| B-A | 0.28186 | 0.20174 | 1.397 | 0.499 |
| C-A | 0.22280 | 0.20935 | 1.064 | 0.710 |
| D-A | 0.63410 | 0.24036 | 2.638 | **0.041** |
| C-B | -0.05905 | 0.20174 | -0.293 | 0.991 |
| D-B | 0.35225 | 0.23376 | 1.507 | 0.431 |
| D-C | 0.41130 | 0.24036 | 1.711 | 0.316 |
|  |  |  |  |  |
| **Metabolism** |  |  |  |  |
| B-A | -0.001 | 0.079 | -0.008 | 1.000 |
| C-A | -0.142 | 0.079 | -1.796 | 0.275 |
| D-A | -0.204 | 0.079 | -2.568 | **0.050** |
| C-B | -0.142 | 0.079 | -1.789 | 0.279 |
| D-B | -0.204 | 0.079 | -2.560 | **0.050** |
| D-C | -0.061 | 0.079 | -0.772 | 0.867 |
